# A Piece of History that Cannot Be Missed for Precision Medicine

**DOI:** 10.1016/j.gpb.2017.01.003

**Published:** 2017-02-05

**Authors:** Jun Yu

**Affiliations:** CAS Key Laboratory of Genome Sciences and Information, Chinese Academy of Sciences, Beijing 100101, China

A couple of years ago when Dr. Maynard V. Olson, my postdoctoral mentor, visited me (Figure 1), it came to my mind that since he is the only person involved in writing the twoUS National Research Council reports [1], [2] that put forward the two landmark projects, the Human Genome Project (HGP) and the Precision Medicine Initiative (PMI), I should dig up more details about how things have come to be, such as the phrase “precision medicine”. I also planned to publish the Chinese translation of the reports and Maynard’s writing will be an excellent introduction to the book [3]. Of course, Maynard agreed and his writing was translated into Chinese and published as Forward in this book. It is unfortunate, however, that the book was only printed 200 copies for its first edition, and it becomes obvious that a very limited number of people may have chance to read it. Maynard’s writing should be read timely by the entire research community of biomedicine. With permission from both Maynard and the publisher, we now publish the original writing in English in *Genomics, Proteomics & Bioinformatics* [4].

The first time when I heard the phrase “precision medicine” was in the evening of May 7, 2012 – less than a year after the publication of the “precision medicine” report but two years before the term becomes popular and the US President Barack Obama’s official announcement of the Initiative. I was on vacation in Seattle and luckily had a chance to enjoy the event. In that evening, Maynard moderated a panel discussion about the future of genome sciences; the panel members include Bruce Alberts (Editor of *Science* and former President of the US National Academy of Sciences), Natalie Angier (a Pulitzer Prize-winning science writer for *The New York Times* and the Andrew D. White Professor-at-Large at Cornell University), James Evans (the Bryson Distinguished Professor of Genetics and Medicine and Director of the Clinical Cancer Genetics Services at University of North Carolina), and Keith Yamamoto (Executive Vice Dean of the School of Medicine, Professor of Cellular and Molecular Pharmacology, and Vice Chancellor for Research at University of California, San Francisco). After the event, I have thoroughly read the report and its ideas immediately won me over. The only thing I was not satisfied with is the network across multiple layers of ever-increasing number of omics. The omics-thinking is rather divergent but what we really need has to be convergent. I have started to formulate some irreducible tracks with distinct functionality lying between genotypes and phenotypes [5] and came up with five of them – informational, operational, homeostatic, compartmental, and plastic, following my earlier thoughts for a new synthesis [6].

I should stop writing before our readers lose their appetite for Maynard’s masterpiece. So, take a deep breath and enjoy Maynard’s story!

## Competing interests

The author has declared no competing interests.

## Figures and Tables

**Figure 1:**
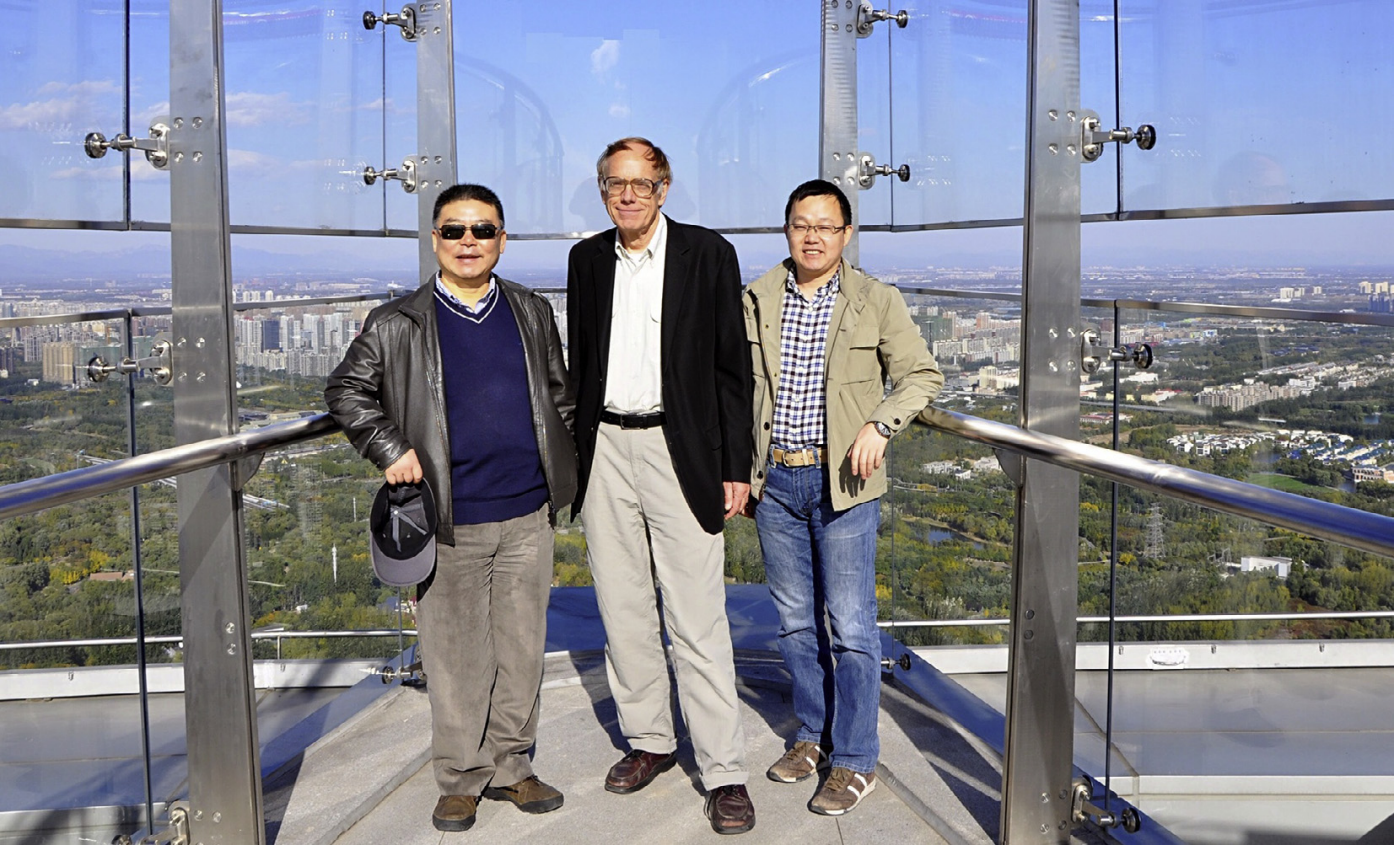
**Jun Yu (left) and Xumin Wang (right) with Maynard V. Olson at Beijing Olympic Tower, Beijing, 2015**
